# Efficacy analysis of comprehensive rehabilitation therapy based on the Gesell developmental schedules among infants with global developmental delay of different ages: a retrospective study

**DOI:** 10.3389/fneur.2025.1568643

**Published:** 2025-07-02

**Authors:** Dan Liu, Mo Duan, Cuiying Chen, Jianhua Xue, Ling Yue

**Affiliations:** ^1^Hebei Provincial Clinical Research Center for Child Health and Disease, Hebei Children’s Hospital, Shijiazhuang, China; ^2^Clinical Laboratory, The Second Honorary Soldier Preferential Treatment Hospital of Hebei Province, Shijiazhuang, China

**Keywords:** global developmental delay, infant patient, Gesell developmental schedules, developmental quotient, early intervention

## Abstract

**Objective:**

This study aimed to analyze differences in efficacy using the Gesell Developmental Schedules (GDS) among infant patients with global developmental delay (GDD), thereby providing an objective basis for early intervention.

**Methods:**

A retrospective analysis was performed of 155 infants with GDD who were first diagnosed and admitted to the neurorehabilitation department of our hospital between January 2022 and December 2024. The data collected included general information, maternal pregnancy and perinatal data, and GDS results of the patients. The patients were divided according to their age into 3–6, 7–12, and 13–18-month groups. All patients received at least 3 months of comprehensive rehabilitation therapy, and the GDS was used to assess pre- and post-treatment efficacy.

**Results:**

According to the logistic regression analysis, age at initial diagnosis, prematurity, and neonatal asphyxia were identified as risk factors for poor prognosis (OR > 1, *p* < 0.05). The pre- and post-treatment difference in the total developmental quotient (DQ) of the five domains (adaptive, gross motor, fine motor, language, and personal-social) were significantly higher in the 3–6-month group than in the 7–12- and 13–18-month groups (*p* < 0.05) and did not differ significantly from the pre-post difference in total DQ between the 7–12- and 13–18-month groups (*p* > 0.05). The 3–6-month group had significantly higher pre-post DQ differences in the five domains than the 7–12- and 13–18-month groups (*p* < 0.05). The 7–12- and 13–18-month groups did not differ significantly with respect to pre-post DQ differences in any of the five domains (*p* > 0.05). Regarding the levels of the GDS adaptive domain, the number of cases at each level differed significantly before and after treatment in the 3–6-month group (*p* < 0.05).

**Conclusion:**

Comprehensive rehabilitation interventions showed significant efficacy in infants aged 3–6 months.

## 1 Background

Global developmental delay (GDD) is a common neurodevelopmental disorder in children with a reported incidence of 1–3% in the global population (1). According to the Diagnostic and Statistical Manual of Mental Disorders, Fifth Edition (DSM-5), revised by the American Psychiatric Association in 2013 (2–4), GDD is usually diagnosed in children under the age of five (5, 6) who exhibit significant delays in two or more developmental milestones (adaptive, gross motor, fine motor, language, and personal-social) compared to the predicted level of their peers; its developmental level should be at least two standard deviations below the mean of the standardized test. GDD is a transitional diagnosis that serves as an early diagnostic criterion for infants who exhibit high-risk factors for cerebral palsy or brain damage. Early detection of this disease, coupled with comprehensive rehabilitation therapy, can greatly reduce the incidence of intellectual developmental disorders and cerebral palsy (7).

The etiology of GDD includes both genetic and non-genetic factors. In this study, 10 children underwent whole-exome sequencing of their families. Among them, three cases had pathogenic mutations: (1) *DYRK1A*, chromosome location: chr21:38862534, variant site: c.722 T > C (p. L241P), associated disease: autosomal dominant intellectual disability type 7; (2) *HUWE1*, chromosome location: chrX:53561008, variant site: c.12982G > C (p. D4328H), associated disease: Turner-type X-linked intellectual developmental disorder; and (3) *POGZ*, chromosome location: chr1:151378587–151378588, variant site: c.2908_2923dup (p. Val975GlufsTer25), associated disease: autosomal dominant intellectual disability type 37.

The Gesell Developmental Schedules (GDS) was developed by American child psychologist Gesell et al.,in the 1940s. It is used to measure the neurodevelopmental level of children under the age of five, as well as to diagnose and assess children who may have developmental delays. It is regarded as a relatively comprehensive diagnostic tool for child development (8). After China conducted norm standardization studies on the GDS for children aged 0–3 and 3.5–6 in 1985 and 1990 respectively, the Beijing Gesell Development Schedules (BGDS) has been widely used in child development assessments. Xi et al. employed the BGDS to assess the developmental level of children with GDD (9). In the present study, we employed the BGDS, aimed to explore the timing and efficacy of rehabilitation therapy in infants (aged 3–18 months) with GDD.

## 2 Methods

### 2.1 Patients

The inclusion criteria were as follows: (1) patients who met the diagnostic criteria of DSM-5 (2013) of the American Psychiatric Association; (2) aged between 3 and 18 months; (3) the presence of developmental milestone delays in two or more domains, on the BGDS, DQ ≤ 75; and (4) whose guardians were willingness to undergo the trial protocol and voluntarily provide written informed consent.

The exclusion criteria were as follows: (1) diagnosis of cerebral palsy, autism spectrum disorder, or other neurodevelopmental disorders; (2) diagnosis of inherited metabolic diseases, congenital myopathies, or peripheral neuropathies; (3) patients with significant auditory or visual impairments that precluded the completion of the assessment; and (4) patients who did not receive effective comprehensive rehabilitation therapy for 3 months or did not complete two rounds of the GDS assessment as scheduled.

**Figure 1:**
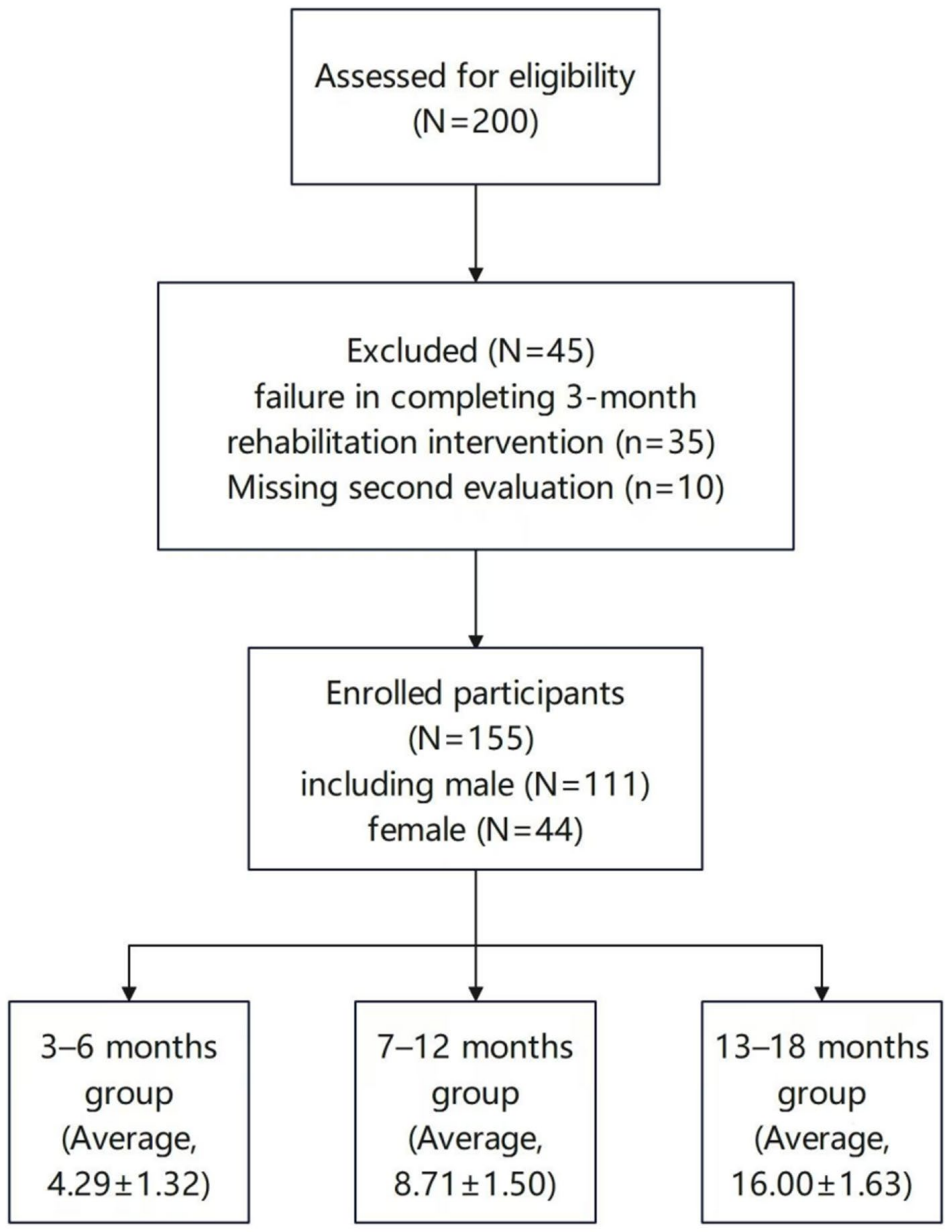
Flowchart of pediatric patients enrollment and stratification by month age.

In this study, 155 children with GDD who met the above criteria and had their first visit to the Department of Neurorehabilitation in our hospital between January 2022 and December 2024 were selected (Figure 1). Data collected from patients with GDD included sex, age (in months), gestational age at birth, birth weight, maternal diseases during pregnancy, perinatal adverse events, neonatal diseases, and GDS results. The baseline characteristics did not differ significantly across the three groups (Table 1). This study was performed with the consent of the patient’s parents, who provided written informed consent and was approved by the Ethics Committee of our hospital (No.: 202407–41).

**Table 1:** Baseline characteristics of the three age groups.

Groups						
Variable		3–6 months	7–12 months	13–18 months	Statistic value	*p-*value
Sex [cases (%)]	Male	58 (74.36)	33 (71.74)	20 (64.52)	*χ^2^* = 1.058	0.589
	Female	20 (25.64)	13 (28.26)	11 (35.48)		
Prematurity [cases (%)]	Yes	25 (32.05)	14 (30.43)	8 (25.81)	*χ^2^* = 0.410	0.815
	No	53 (67.95)	32 (69.57)	23 (74.19)		
Neonatal asphyxia[cases (%)]	Yes	21 (26.92)	16 (34.78)	12 (38.71)	*χ^2^* = 1.729	0.421
	No	57 (73.08)	30 (65.22)	19 (61.29)		
Birth weight(kg)		2.76 ± 0.734	2.79 ± 0.843	2.98 ± 0.747	*F* = 0.936	0.395
DQ (score, –x ± s)	Adaptive	59.32 ± 19.835	63.89 ± 14.433	56.94 ± 14.895	*F* = 1.669	0.192
	Gross motor	60.86 ± 16.930	61.83 ± 14.622	54.94 ± 13.672	*F* = 2.050	0.132
	Fine motor	64.56 ± 18.617	68.87 ± 15.430	60.55 ± 14.139	*F* = 2.308	0.103
	Language	63.59 ± 19.662	67.70 ± 15.008	59.77 ± 13.861	*F* = 1.988	0.140
	Personal-social	58.83 ± 17.807	65.39 ± 14.290	59.10 ± 12.668	*F* = 2.692	0.071

### 2.2 Neurodevelopmental assessment

Assessments were performed before treatment and 3 months after treatment using the Chinese version of the GDS (revised by the Beijing Children’s Hospital Healthcare Center). The assessment was performed by professionally trained rehabilitation therapists, and the results were reviewed by the associate chief physicians. The assessment involved evaluating the developmental quotient (DQ) across five domains: adaptive, gross motor, fine motor, language, and personal-social. The DQ of each domain was defined as 100 × (developmental age/actual age) (10). The assessment results were divided into six levels: DQ > 85, normal; 76–85, borderline; 55–75, mild; 40–54, moderate; 25–39, severe; and < 25, indicating extremely severe developmental delay.

### 2.3 Comprehensive rehabilitation training

**Figure 2:**
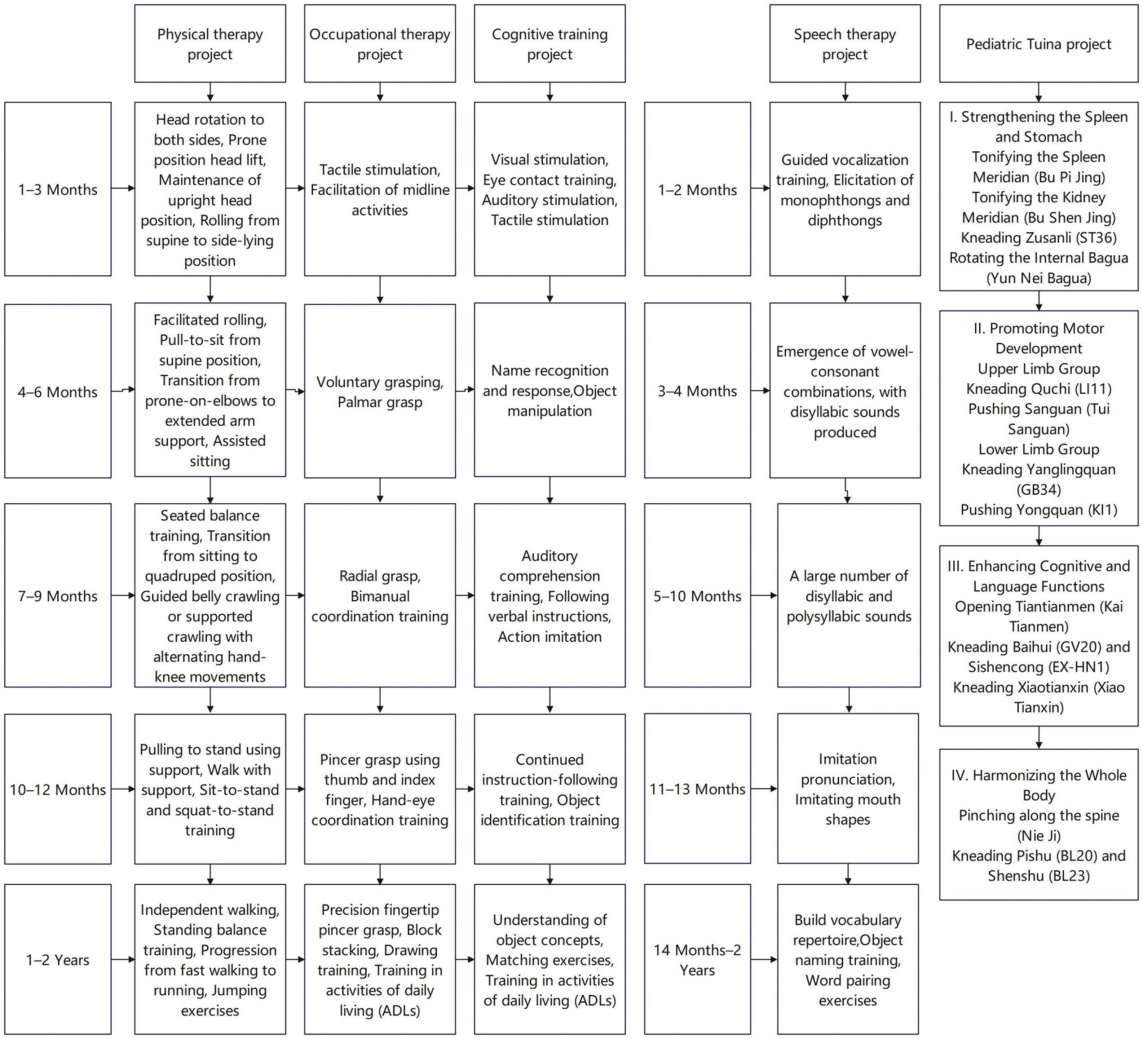
Comprehensive rehabilitation training program.

All patients received comprehensive rehabilitation training, including exercise therapy, occupational therapy, cognitive training, speech therapy, and pediatric Tuina (11–13), as shown in (Figure 2). Once daily for 30 min per session, five times per week. The patients were hospitalized for at least three consecutive months,

### 2.4 Prognostic evaluation

A post-treatment Adaptive Behavior Domain DQ score greater than 85 on the GDS was considered indicative of a favorable prognosis, whereas a score of 85 or less indicated a poor prognosis.

### 2.5 Statistical analysis

Data analysis was performed using the statistical software IBM SPSS (version 24.0; IBM Corp., Armonk, NY, United States). The Shapiro–Wilk test was used to assess the normality of continuous data, Normally distributed measurement data are expressed as ^−^*x* ± *s*, and comparisons among multiple groups were performed using one-way analysis of variance. Count data are expressed as the number of cases and percentages, The chi-square test was used for categorical variables, and the rank-sum test was used for ordinal data. Logistic regression analysis was performed to identify factors influencing the prognosis of children with GDD. *p* < 0.05 was considered statistically significant.

## 3 Results

### 3.1 Etiology analysis of patients with GDD

After screening for the main influencing factors using univariate logistic regression analysis, significant variables were included in a multivariate logistic regression model. The results indicated that age at initial diagnosis, prematurity, and neonatal asphyxia were risk factors for poor prognosis in children with GDD (OR > 1, *p* < 0.05), as shown in(Table 2).

**Table 2:** Analysis of factors influencing poor prognosis in patients with GDD.

Influencing factors	*β*	SE	Wald *χ*^2^ value	OR	95% CI	*P-*value
Age at first diagnosis	0.149	0.055	7.245	1.161	1.041–1.293	0.007
Prematurity	0.957	0.481	3.953	2.604	1.014–6.687	0.047
Neonatal asphyxia	0.949	0.463	4.196	2.583	1.042–6.404	0.041
Bilirubin encephalopathy	0.077	0.486	0.025	1.080	0.417–2.798	0.874
Neonatal HIE	0.262	0.499	0.277	1.300	0.489–3.455	0.599

### 3.2 Comparison of pre-post DQ difference in the five domains among the three age groups

The total DQ of the five domains after treatment was subtracted from the total DQ before treatment to obtain the total DQ difference. A comparison of the total DQ differences revealed statistically significant differences across the three age groups (*p* = 0.001). More specifically, the pre-to post-treatment difference in total DQ of the 3–6-month group was significantly higher than that of the 7–12- and 13–18-month groups (*p* = 0.002 and 0.001, respectively), while the 7–12- and 13–18-month groups did not differ significantly with respect to the pre-to post-treatment difference in total DQ (*p* = 0.507).

The individual DQ of the five domains were selected, and the post-treatment DQ was subtracted from the pre-treatment DQ to obtain the difference in DQ. Comparison of pre-to-post treatment differences in DQ indicated that the 3–6-month group had significantly higher DQ differences in the five domains (adaptive, gross motor, fine motor, language, and personal-social) compared to both the 7–12-month group (*p* = 0.010, 0.019, 0.013, 0.033, and 0.021, respectively) and 13–18-month group (*p* = 0.015, 0.006, 0.031, 0.038, and <0.001, respectively). The 7–12- and 13–18-month groups did not differ significantly with respect to the DQ differences in all five domains (*p* = 0.856, 0.530, 0.987, 0.850, and 0.082 for adaptive, gross motor, fine motor, language, and personal-social, respectively; Table 3).

**Table 3:** Comparison of pre-post DQ difference in the five domains among the three age groups.

Groups					
Variable	3–6 months	7–12 months	13–18 months	Statistic value	*p-*value
Total DQ difference	61.40 ± 68.526	27.74 ± 51.806	18.71 ± 35.057	*F* = 8.109	<0.001
Adaptive DQ difference	15.03 ± 17.277	7.35 ± 16.508	6.68 ± 10.480	*F* = 4.843	0.009
Gross motor DQ difference	10.41 ± 15.294	4.48 ± 10.909	2.52 ± 11.601	*F* = 5.053	0.008
Fine motor DQ difference	10.72 ± 16.802	3.72 ± 15.073	3.77 ± 8.850	*F* = 4.179	0.017
Language DQ difference	10.59 ± 19.277	3.61 ± 16.919	2.84 ± 12.877	*F* = 3.387	0.036
Personal-social DQ difference	14.65 ± 16.091	8.59 ± 12.729	2.90 ± 8.960	*F* = 8.471	<0.001

### 3.3 Comparison of pre-post levels in the GDS adaptive domain among the three age groups

A significant difference in the pre- and post-treatment levels of the adaptive domain was observed in the 3–6-month group (*p* < 0.001). However, this difference was not significant in the 7–12- and 13–18-month groups (*p* > 0.05; Table 4).

**Table 4:** Comparison of pre-post levels in the GDS adaptive domain among the three age groups.

Level	3–6 months (78 cases)		7–12 months (46 cases)		13–18 months (31 cases)	
	Pre-treatment (%)	Post-treatment (%)	Pre-treatment (%)	Post-treatment (%)	Pre-treatment (%)	Post-treatment (%)
Normal	7 (9.0)	27 (34.6)	0 (0)	8 (17.4)	1 (3.7)	3 (9.7)
Borderline	10 (12.8)	18 (23.1)	13 (28.2)	10 (21.7)	0 (0)	6 (19.4)
Mild	32 (41.0)	25 (32.1)	20 (43.5)	15 (32.6)	16 (51.9)	13 (41.9)
Moderate	16 (20.5)	4 (5.1)	11 (23.9)	11 (23.9)	10 (29.6)	7 (22.6)
Severe	8 (10.2)	3 (3.8)	2 (4.3)	1 (2.2)	4 (14.8)	2 (6.5)
Extremely severe	5 (6.4)	1 (1.3)	0 (0)	1 (2.2)	0 (0)	0 (0)
Statistic value	Z = -4.569		Z = -1.021		Z = -1.379	
*p*-value	<0.001		0.307		0.168	

## 4 Discussion

GDD is an early-onset neurological disease commonly found in infants and young children and is a major cause of childhood disability worldwide (14, 15). This disease can have a lifelong impact on the patient’s quality of life and may lead to lifelong disability in severe cases. Therefore, analyzing the efficacy of early interventions for patients with GDD and determining the prognosis of their neurobehavioral development has significant implications for improving the patient’s quality of life and enhancing population quality, thereby alleviating the burden on family and society.

Patients with GDD exhibit a complex and diverse etiology, and the results indicate that age at initial diagnosis, prematurity, and neonatal asphyxia are risk factors. Age at initial diagnosis indicating that with advancing age, the risk of poor prognosis increases. This highlights that early diagnosis is a crucial aspect for the prognosis of GDD. In this study, among preterm infants, one third were very preterm and extremely preterm. Younger gestational age is associated with a higher likelihood of severe asphyxia (16). The reason may be that preterm infants’ organs are still immature, with insufficient pulmonary surfactant and weak respiratory muscles. Advances in perinatal medicine and neonatal rescue techniques have contributed to an increase in the survival rate of extremely premature infants, as well as a gradual rise in the prevalence rate of developmental disorders (17–19). Therefore, emphasis should be placed on early intervention and treatment to improve the patient’s quality of life (20–22).

Previous studies have found that GDD occurs more frequently in males than females (23). Similarly, we found a higher number of male patients in the study population (71.6%). Cuppens et al. found that sex influenced the developmental aspects of gene expression for GDD-associated genes mutated in males and females in the dorsolateral prefrontal cortex and posterior (caudal) superior temporal cortex (area 22c) brain regions, which could also explain differences in clinical manifestations (4). This suggests that greater attention should be paid to early assessment of high-risk factors in male infants.

However, the brains of infants and young children are not fully mature. According to the theory of neuroplasticity, younger individuals have greater plasticity, which gradually decreases with age (24, 25). Our findings revealed that the total DQ of the five domains showed an increase after treatment in patients with GDD, while the improvements in the results for the five domains were consistent with those of the total DQ difference, with infants aged 3–6 months showing the most significant improvement. The reason may be brain development occurs most rapidly during the first 6 months of life. During this period of rapid brain development, exposure to multisensory stimulation (26), including touch, hearing, sight, vestibular sense, can promote axonal regeneration, remyelination, synaptic connection, and neural reorganization. Tang et al. showed that infants at high risk of brain damage should receive early intervention, and the “golden period” for early intervention is within the first 4 months. Therefore, we propose that the time window for GDD rehabilitation intervention should be moved forward within the first 6 months.

The efficacy of comprehensive rehabilitation intervention for infants with GDD is not only reflected in the increase in DQ but also verified by the change in severity level. The adaptive domain is a key domain in BGDS. It’s a child’s ability to organize objects, perceive interrelationships, and solve problems reflects foundational cognitive skills that serve as precursors to future intelligence. Patients in the 3–6-month group showed a significant difference in the severity level after comprehensive rehabilitation intervention. This further demonstrates the theory of neuroplasticity, Furthermore, treatment response demonstrates age-dependent enhancement, peaking in the youngest cohort. Level changes were mostly concentrated among cases that improved by one level, remained unchanged, or improved by two levels. The decrease in levels may have been because these children suffered from severe brain damage and were unable to attain the developmental speed of healthy children despite the rehabilitation intervention. This led to a widening gap between them and healthy children, causing their scores to gradually decrease. However, these patients still showed gradual functional progress compared with their baseline values.

Our study had some limitations. First, the sample size was insufficient, and the observation time was short, which may have affected the statistical results. Second, the results of the GDS were observed only up to 18 months of age. Thus, further follow-up investigations are required to assess the neurodevelopmental status of infants with GDD.

## 5 Conclusion

Comprehensive rehabilitation outcomes across three cohorts stratified by chronological age, Infant patients aged 3–6 months showed the highest efficacy. Infants with high-risk factors such as prematurity or perinatal asphyxia require heightened vigilance and regular developmental surveillance. Early diagnosis and early intervention are crucial factors in reducing the disability rate among infants with GDD.

## References

[1] Lin L Zhang Y Pan H Wang J Qi Y Ma Y. Clinical and genetic characteristics and prenatal diagnosis of patients presented GDD/ID with rare monogenic causes. Orphanet J Rare Dis. (2020) 15:317. doi: 10.1186/s13023-020-01599-y, PMID: 33176815 PMC7656751

[2] Shan L Feng JY Wang TT Xu ZD Jia FY. Prevalence and developmental profiles of autism spectrum disorders in children with global developmental delay. Front Psych. (2021) 12:794238. doi: 10.3389/fpsyt.2021.794238, PMID: 35115968 PMC8803654

[3] Al-Yamani AI Sulaiman NA Al-Ansari AM. An evaluation of the effectiveness of a therapeutic program for children with global developmental delay. Arab Gulf J Sci Res. (2023) 41:627–37. doi: 10.1108/agjsr-09-2022-0195

[4] Cuppens T Shatto J Mangnier L Kumar AA Ng AC Kaur M. Sex difference contributes to phenotypic diversity in individuals with neurodevelopmental disorders. Front Pediatr. (2023) 11:1172154. doi: 10.3389/fped.2023.1172154, PMID: 37609366 PMC10441218

[5] Choo YY Agarwal P How CH Yeleswarapu SP. Developmental delay: identification and management at primary care level. Singapore Med J. (2019) 60:119–23. doi: 10.11622/smedj.2019025, PMID: 30997518 PMC6441684

[6] Shchubelka K Turova L Wolfsberger W Kalanquin K Williston K Kurutsa O. Genetic determinants of global developmental delay and intellectual disability in Ukrainian children. J Neurodev Disord. (2024) 16:13. doi: 10.1186/s11689-024-09528-x, PMID: 38539105 PMC10967201

[7] Jeong J Franchett EE Ramos de Oliveira CV Rehmani K Yousafzai AK. Parenting interventions to promote early child development in the first three years of life: a global systematic review and meta-analysis. PLoS Med. (2021) 18:e1003602. doi: 10.1371/journal.pmed.1003602, PMID: 33970913 PMC8109838

[8] Chen G Tan W Shi Y Chen M Huang Q Lin Y. On Gesell evaluation system for disabled children in minority areas. J Healthc Eng. (2022) 2022:4210116. doi: 10.1155/2022/4210116, PMID: 35126922 PMC8808213

[9] Fei X Song Y Yan S Long X Liang A Wang Y. Relationship between fundamental motor skills and physical fitness in children with global developmental delay. Pediatr Investig. (2024) 8:201–8. doi: 10.1002/ped4.12452, PMID: 39347524 PMC11427902

[10] Tian W Zhao X Xu H Sun Y Zhu M. Application of the Hammersmith infant neurological examination in the developmental follow‐up of high‐risk infants. Dev Med Child Neurol. (2024) 66:1181–9. doi: 10.1111/dmcn.15855, PMID: 38308400

[11] Juneja M Gupta A Sairam S Jain R Sharma M Thadani A. Diagnosis and Management of Global Development Delay: consensus guidelines of growth, development and behavioral pediatrics chapter, neurology chapter and neurodevelopment pediatrics chapter of the Indian academy of pediatrics. Indian Pediatr. (2022) 59:401–15. doi: 10.1007/s13312-022-2522-5, PMID: 35188106

[12] Ankar P Sharath HV Chavan N. A case report of pediatric rehabilitation for hypoxic ischemic encephalopathy associated with global developmental delay. Cureus. (2024) 16:e54851. doi: 10.7759/cureus.54851, PMID: 38533149 PMC10964207

[13] Li F Tian J Yuan F Zhao W Chen H Hao J. Efficacy of early clinical interventions for children with global developmental delay. Int J Neurosci. (2025) 135:280–6. doi: 10.1080/00207454.2023.2298715, PMID: 38284177

[14] Liao LH Chen C Peng J Wu LW He F Yang LF. Diagnosis of intellectual disability/global developmental delay via genetic analysis in a central region of China. Chin Med J. (2019) 132:1533–40. doi: 10.1097/CM9.0000000000000295, PMID: 31205075 PMC6616229

[15] Palumbo P Di Muro E Accadia M Benvenuto M Di Giacomo MC Castellana S. Whole exome sequencing reveals a novel AUTS2 in-frame deletion in a boy with global developmental delay, absent speech, dysmorphic features, and cerebral anomalies. Genes (Basel). (2021) 12:12020229. doi: 10.3390/genes12020229, PMID: 33562463 PMC7915150

[16] Ahmed R Mosa H Sultan M Helill SE Assefa B Abdu M. Prevalence and risk factors associated with birth asphyxia among neonates delivered in Ethiopia: a systematic review and meta-analysis. PLoS One. (2021) 16:e0255488. doi: 10.1371/journal.pone.0255488, PMID: 34351953 PMC8341515

[17] Hee Chung E Chou J Brown KA. Neurodevelopmental outcomes of preterm infants: a recent literature review. Transl Pediatr. (2020) 9:S3–8. doi: 10.21037/tp.2019.09.10, PMID: 32206579 PMC7082240

[18] Wang LW Chu CH Lin YC Huang CC. Severe brain injury and trends of gestational-age-related neurodevelopmental outcomes in infants born very preterm: a population cohort study. Dev Med Child Neurol. (2025) 67:59–67. doi: 10.1111/dmcn.16003, PMID: 38946133

[19] Camerota M Lester BM. Neurobehavioral outcomes of preterm infants: toward a holistic approach. Pediatr Res. (2024) 97:305. doi: 10.1038/s41390-024-03505-9, PMID: 39179875 PMC11846960

[20] Zhang J Xu Y Liu Y Yue L Jin H Chen Y. Genetic testing for global developmental delay in early childhood. JAMA Netw Open. (2024) 7:e2415084. doi: 10.1001/jamanetworkopen.2024.15084, PMID: 38837156 PMC11154162

[21] Kim SW Jeon HR Jung HJ Kim JA Song JE Kim J. Clinical characteristics of developmentally delayed children based on interdisciplinary evaluation. Sci Rep. (2020) 10:8148. doi: 10.1038/s41598-020-64875-8, PMID: 32424178 PMC7235222

[22] Okwara FNO le Roux SM Donald KA. Health service utilization by young children with autism Spectrum disorder versus global developmental delay at a tertiary Center in a Resource-Limited Setting. J Dev Behav Pediatr. (2022) 43:e320–9. doi: 10.1097/DBP.0000000000001034, PMID: 35125466

[23] Randhawa HS Bagale S Umap R Randhawa J. Brain magnetic resonance imaging-based evaluation of pediatric patients with developmental delay: a cross-sectional study. Cureus. (2022) 14:e24051. doi: 10.7759/cureus.24051, PMID: 35573542 PMC9095436

[24] Finch-Edmondson M Morgan C Hunt RW Novak I. Emergent prophylactic, reparative and restorative brain interventions for infants born preterm with cerebral palsy. Front Physiol. (2019) 10:15. doi: 10.3389/fphys.2019.00015, PMID: 30745876 PMC6360173

[25] Festante F Antonelli C Chorna O Corsi G Guzzetta A. Parent-infant interaction during the first year of life in infants at high risk for cerebral palsy: a systematic review of the literature. Neural Plast. (2019) 2019:5759694. doi: 10.1155/2019/5759694, PMID: 31178902 PMC6501141

[26] Lazarus MF Marchman VA Brignoni-Perez E Dubner S Feldman HM Scala M. Inpatient skin-to-skin care predicts 12-month neurodevelopmental outcomes in very preterm infants. J Pediatr. (2024) 274:114190. doi: 10.1016/j.jpeds.2024.114190, PMID: 39004169 PMC11514444

